# Efficacy of an insecticide paint against insecticide-susceptible and resistant mosquitoes - Part 1: Laboratory evaluation

**DOI:** 10.1186/1475-2875-9-340

**Published:** 2010-11-25

**Authors:** Beatriz Mosqueira, Stéphane Duchon, Fabrice Chandre, Jean-Marc Hougard, Pierre Carnevale, Santiago Mas-Coma

**Affiliations:** 1 Departamento de Parasitología, Facultad de Farmacia, Universidad de Valencia, Av. Vicent Andrés Estellés s/n, 46100 Burjassot, Valencia, Spain; 2 UR016, Institut de Recherche pour le Développement (IRD), BP 64501, 34394 Montpellier Cedex 5, France

## Abstract

**Background:**

The main malaria vector *Anopheles gambiae *and the urban pest nuisance *Culex quinquefasciatus *are increasingly resistant to pyrethroids in many African countries. There is a need for new products and strategies. Insecticide paint Inesfly 5A IGR™, containing two organophosphates (OPs), chlorpyrifos and diazinon, and insect growth regulator (IGR), pyriproxyfen, was tested under laboratory conditions for 12 months following WHOPES Phase I procedures.

**Methods:**

Mosquitoes used were laboratory strains of *Cx. quinquefasciatus *susceptible and resistant to OPs. The paint was applied at two different doses (1 kg/6 m^2 ^and 1 kg/12 m^2^) on different commonly used surfaces: porous (cement and stucco) and non-porous (softwood and hard plastic). Insecticide efficacy was studied in terms of delayed mortality using 30-minute WHO bioassay cones. IGR efficacy on fecundity, fertility and larval development was studied on OP-resistant females exposed for 30 minutes to cement treated and control surfaces.

**Results:**

After treatment, delayed mortality was high (87-100%) even against OP-resistant females on all surfaces except cement treated at 1 kg/12 m^2^. Remarkably, one year after treatment delayed mortality was 93-100% against OP-resistant females on non-porous surfaces at both doses. On cement, death rates were low 12 months after treatment regardless of the dose and the resistance status. Fecundity, fertility and adult emergence were reduced after treatment even at the lower dose (p < 10^^-3^^). A reduction in fecundity was still observed nine months after treatment at both doses (p < 10^^-3^^) and adult emergence was reduced at the higher dose (p < 10^^-3^^).

**Conclusions:**

High mortality rates were observed against laboratory strains of the pest mosquito *Cx. quinquefasciatus *susceptible and resistant to insecticides. Long-term killing remained equally important on non-porous surfaces regardless the resistance status for over 12 months. The paint's effect on fecundity, fertility and adult emergence may continue to provide an additional angle of attack in reducing overall population densities when the lethal effect of OPs diminishes over time. Some options on how to deal with porous materials are given. Implications in vector control are discussed.

## Background

Every year, 300-500 million clinical episodes of malaria occur, resulting in about one million deaths [1]. A vast majority of these deaths involve children less than 5 years old in sub-Saharan Africa [2,3]. The fighting of malaria in sub-Saharan Africa is mainly focused on vector control through the use of insecticide-treated nets (ITNs) and indoor residual insecticide spraying (IRS) [4,5]. At present, pyrethroids are the only insecticides recommended for treatment of mosquito nets [6]. Despite the great value of pyrethroid-treated nets in malaria vector control, their efficacy may be threatened by resistance of major malaria vectors to this class of insecticides [7]. IRS is the main method of attacking adult mosquitoes in houses, but the technique requires teams of trained personnel and special equipment to be transported to where they are needed [8].

A present recommendation towards resistance management is alternating or using in combination different insecticides or novel strategies in the framework of an integrated vector management [9] while respecting the Stockholm Convention on Persistent Organic Pollutants (POPs) of finding sustainable alternatives to POPs in integrated pest management practices where possible [10].

Insecticide paint Inesfly 5A IGR™ is composed of two organophosphates (OPs), chlorpyriphos (1.5%) and diazinon (1.5%) and an insect growth regulator (IGR), pyriproxyfen (0,063%). The product is a white vinyl paint with an aqueous base. Active ingredients reside within Ca CO^3 ^+ resin microcapsules. The formulation allows a gradual release of active ingredients, increasing its durability. Microcapsules range from one to several hundred micrometers in size. Toxicology studies performed so far support the paint's safety in terms of irritancy (ocular, dermal and systemic), cytotoxicity and mutagenicity [11] and allergenicity [12]. Acute inhalation toxicity studies classified this paint as Category III (according to WHO) and category IV (according to EPA) - no warning label required in either case [12]. Analysis on cholinesterase levels showed no variations before/after treatment. Values were within reference values for all subjects [13].

The efficacy of Inesfly 5A IGR™ was studied under laboratory conditions for over 12 months at the *Laboratoire de Lutte contre les Insectes Nuisibles/Institut de Recherche pour le Développement *(LIN/IRD), the WHO reference laboratory for insecticide testing, in Montpellier, France. These highly-controlled evaluations against mosquitoes specifically resistant to the paint's insecticides were triggered by the encouraging results obtained during preliminary testing in malaria-endemic areas in Benin and Côte d'Ivoire, West Africa, against local populations of *Anopheles gambiae *the main malaria vector in sub-Saharan Africa. Resistance to OPs has been described in vector and pest mosquitoes in various parts of the world, including West Africa [14,15]. The paint's efficacy was tested against laboratory strains of the urban pest *Culex quinquefasciatus *susceptible and resistant to OPs. At the time of the study, there was no laboratory strain of *An. gambiae *specifically resistant to OPs. Efficacy was studied in two ways: delayed mortality and effect of the IGR on fecundity, fertility and larval development.

## Methods

### Delayed mortality

30 minute-WHO bioassay cones [16] were performed against two laboratory strains of *Cx. quinquefasciatus*: *Cx. quinquefasciatus *S-Lab is an insecticide susceptible reference strain [17]. *Culex.quinquefasciatus *SR is homozygote for the ace-1^*R *^resistant gene involved in the resistance to OPs and carbamates, but has the same genetic background as S-Lab [18]. Unfed, 3-5 day old females bred at the LIN insectarium were placed in forced contact with four different surfaces: softwood and hard plastic (non-porous materials) and ready-mixed cement and ready-mixed stucco (porous materials). There were two treated and two controls for each kind of surface. Treated surfaces were painted at two doses, 1 kg/6 m^2 ^(manufacturer's recommended dose to leave surfaces completely white) and 1 kg/12 m^2^. For each kind of surface, one control was left untreated and the other one was painted at 1 kg/6 m^2 ^with the same paint but without the insecticides and the IGR. Paint was applied undiluted with a regular brush and left to dry for 48 hours. After a 30-minute exposure, mosquitoes were introduced in 150-ml plastic cups provided with honey-juice. Tests were done in four repeats using 15 females per cone. Females were left at a temperature of 27 ± 1°C and a relative humidity of 80%, for 24-hour delayed mortality assessments. Tests were done at intervals of six months for one year. When not tested, surfaces were stored in aluminium foil at a temperature of 27 ± 1°C and a relative humidity of 80%. Delayed mortality was analysed using Epi-Info 6. Where values were <5, Fisher exact tests were used. Because bioassay tests are subject to variations, a 99% confidence interval was applied.

### IGR efficacy on fecundity, fertility and larval development

Females were 4-5 day-old to increase the probability of having had females fertilised by male mosquitoes. LIN-reared *Cx. quinquefasciatus *OP-resistant females were exposed to treated and control surfaces for 30 minutes. Females alive 24 hours after a 30-minute exposition, were put in cages and allowed to blood-feed for one night. Females that had been well blood-fed were put in a new cage and given honey-juice every two days. At T0, 50 blood fed females were tested per surface. At T9, 30 blood-fed females were tested per surface. At T0 and T9, blood feeding took place about 36 hours after previous exposition to treated or control surfaces. Efficacy was measured in terms of fecundity (number of eggs laid), fertility (% hatching) and larval development (% pupation and % emergence). Tests were not done using susceptible *Cx. quinquefasciatus *S-Lab, because they all died during the 30-minute exposition. Tests were carried out on the most porous surface, cement, because not enough females survived exposition to other surfaces. Eggs were counted with a dissecting microscope and placed in plastic measuring containers with 2L of water for hatching. Water loss due to evaporation was replaced daily. Larvae were fed every two days. The mean number of eggs was compared between treated and non-treated surfaces using a student T test. Differences in % hatching, % pupation, and % emergence were analysed using Epi-Info 6. Where values were <5, Fisher exact tests were used.

## Results

### Delayed mortality

Delayed 24-hour mortality at T0 was 98-100% (compared to control, p < 10^-3^) for both, susceptible S-Lab and OP-resistant *Cx. quinquefasciatus *on non-porous surfaces and porous surfaces treated at 1 kg/6 m^2^. While non-porous surfaces performed equally well regardless the dose and the resistance status, porous surfaces, cement and stucco, treated at the lower dose 1 kg/12 m^2 ^performed less optimally against OP-resistant mosquitoes yielding mortalities of 87% (p < 10^-3^) and 15% (p < 10^-2^) respectively. Efficacy had dropped by six months on cement surfaces treated at both doses on resistant and susceptible mosquitoes while, on stucco, only OP-resistant *Cx. quinquefasciatus *experienced a drop. Mortality at 24 hours was of 90-100% (compared to control, p < 10^-3^) 12 months after treatment even against resistant mosquitoes at the lower dose on all but porous surfaces (Table 1).

**Table 1:** Delayed 24-hour mortality rates of susceptible *Cx. quinquefasciatus *S-Lab and OP-resistant *Cx. quinquefasciatus *after a 30-minute exposure to control and Inesfly(r) treated surfaces using WHO bioassay cones.

*Culex *% Delayed mortality 24 h (N = 60)	Control 1 No paint	Control 2 Control Paint	Cement 1 Kg/6 m2	Cement 1 Kg/12 m2	Stucco 1 Kg/6 m2	Stucco 1 Kg/12 m2	Softwood 1 Kg/6 m2	Softwood 1 Kg/12 m2	Plastic 1 Kg/6 m2	Plastic 1 Kg/12 m2
			
			Porous Surfaces				Non-Porous Surfaces			
T0 OP-Susceptible	0.5	0.4	100†	100†	100†	100†	100†	100†	100†	100†

**T0 OP-Resistant**	**2**	**2.2**	**100†**	**15.7†**	**100†**	**87.3†**	**100†**	**100†**	**100†**	**100†**

T6 OP-Susceptible	2.2	2.9	3.1	1.7	100†	96.7†	100†	100†	100†	100†

**T6 OP-Resistant**	**1.6**	**3.3**	**0**	**0**	**31.7†**	**3.3**	**100†**	**100†**	**100†**	**100†**

T12 OP-Susceptible	0	2.1	2	0	91.4†	20.3†	100†	100†	100†	100†

**T12 OP-Resistant**	**1.5**	**1**	**4.1**	**5.3**	**20.3†**	**5.3**	**100†**	**93.2†**	**100†**	**100†**

Control 1 = No paint; Control 2 = Paint without insecticide at 1 Kg/6 m^2^. Culex = *Cx. quinquefasciatus*; T0 = 0 months after treatment, T6 = 6 months after treatment, T12 = 12 months after treatment, N = sample size per surface tested; (-) females had already died during the first hour. † = significant differences from control (*P *< 0.05).

### IGR efficacy on fecundity, fertility and larval development

A significant reduction in the number of eggs laid was shown at 0 and 9 months after treatment at either dose (p < 10^^-3^^) (Tables 2 and 3). A reduction in egg hatching was observed at T0 (p < 10^^-3^^), but not at T9. An increased mortality from the nymph to the adult stage was shown 0 months after treatment at the lower dose (p < 10^^-3^^), and 9 months after treatment only at the higher dose (p < 10^^-3^^) No differences were found on the duration of the larval development cycle. No IGR effect was observed 12 months after treatment.

**Table 2:** IGR effect on fecundity, fertility and larval development of females exposed to treated surfaces for 30 minutes.

T0 - Cement (N = 50) OP-resistant *Culex*	Egg number	% Egg-hatching	% Pupation	% Emergence
C1/NO Paint	2104	51.8	39.6	79.5

C2/Paint NO insecticide	2473	48.8	40.0	85.9

Insecticide at 1 Kg/6 m²	No survivors			

Insecticide at 1 Kg/12 m²	800†	41.3†	45.5	52.7†

Control 1 = No paint; Control 2 = Paint without insecticide at 1 Kg/6 m^2^; Culex = *Cx. quinquefasciatus*; T0 = 0 months after treatment; N = sample size per surface tested. † = significant differences from control (*P *< 0.05).

**Table 3:** IGR effect on fecundity, fertility and larval development of females exposed to treated surfaces for 30 minutes.

T9 - Cement (N = 30) OP-resistant *Culex*	Egg number	% Egg-hatching	% Pupation	% Emergence
C1/NO Paint	1908	75.8	56.3	87.8

C2/Paint NO Insecticide	2002	73.1	60.0	84.4

Insecticide at 1 Kg/6 m²	1216†	77.5	64.6	65.9†

Insecticide at 1 Kg/12 m²	1156†	70.9	59.9	86.6

Control 1 = No paint; Control 2 = Paint without insecticide at 1 Kg/6 m^2^; Culex = *Cx. quinquefasciatus*; T9 = 9 months after treatment; N = sample size per surface tested. † = significant differences from control (*P *< 0.05)

## Discussion

After treatment with insecticide paint Inesfly 5A IGR™, 100% of OP-susceptible females died after 24-hours on all surfaces, porous and non-porous at both doses, 1 kg/6 m^2 ^and 1 kg/12 m^2^. Killing was significant (87-100%) even against OP-resistant females on all surfaces except cement treated at the lower dose, 1 kg/12 m^2^.

One year after initial treatment, mortality rates were still quite high, 93-100%, on non-porous surfaces (softwood and hard plastic) at both doses and against both, OP-resistant and susceptible females. On the other hand, the lethal effect on porous surfaces like cement had disappeared by six months after treatment against resistant and susceptible mosquitoes.

Long-term efficacy was an issue of porosity of materials rather than the pH of materials or the dose applied: active principles are kept in an acid pH within its microcapsule, making it more resistant to alkalinity than other conventional paints. To study whether efficacy hinged more on porosity than dose, a parallel study was performed. Cement-made surfaces painted with a control layer and an insecticide paint layer at 1 kg/6 m^2^, performed as well as two insecticide paint layers at 1 kg/6 m^2^, even though the latter had twice the dose (Mosqueira *et al.*, unpublished data). Hence, the first layer acted as a screen (even if it did not have insecticide) that allowed the bioavailability of the insecticide on the second layer. Porosity is also an issue for IRS. DDT may last for six months on cement surfaces, though it usually leaves walls stained [19].

A Phase II field study on this same paint, Inesfly 5A IGR™, was performed in Benin, West Africa for one year against local *An. gambiae *and *Cx. quinquefasciatus *populations resistant to pyrethroids. Experimental houses were built with locally-made cement. Long-term efficacy tests included 30 minute-WHO bioassay cones using the insecticide susceptible reference strain *Cx. quinquefasciatus *S-Lab. Six months after treatment, mortality rates in the Phase II study on cement-made surfaces treated with one layer at 1 Kg/6 m^2 ^were still very high, 98-100% [20] compared to the 3% observed in the Phase I study. The difference observed in the long-term efficacy may be due to the type of cement used in Phase I and II, ready-mixed cement *versus *traditionally made cement, respectively. The greater the proportion of water to cement, the more porous the hardened cement will be. To test this hypothesis, Phase I surfaces with locally made cement were made in Benin. Surfaces were kept away from light when not tested. Temperature and humidity were the same to Phase II experimental houses. Mortality rates were lower on the Phase I Benin surfaces but differences were not significant compared to Phase II cement houses (Mosqueira *et al*., unpublished data) as opposed to the mortality rates obtained on Phase I mixed-cement surfaces.

Another recent study has tested the efficacy and the residual effect of Inesfly 5A IGR™ insecticide paint against the main vector of Chagas disease in South America, *Triatoma infestans*, on different surfaces (wood, cement block and adobe bricks). Insecticide paint yielded longer and higher mortality rates in triatomines than other conventional products [21], and porosity also seemed to be an issue - cement surfaces performed worse than wood and even adobe-made surfaces. Insecticide paints have been used for some time concomitantly with home improvement as a control method for Chagas disease with good results [22,23].

Pyriproxyfen is toxic to a broad spectrum of insects during their developmental stages. Research on the dengue vector, *Aedes aegypti*, shows that contaminated adults can render oviposition sites unproductive by horizontal dissemination of pyriproxyfen even at small concentrations [24-26]. A study performed by Itoh *et al *[24] showed pyriproxyfen had a larger impact on fecundity when females were exposed to pyriproxyfen before blood feeding. Inversely, pyriproxyfen's effect on egg-hatching [24,27,28] and adult emergence [24,28] seems to be higher when females have blood fed before being exposed to treatment.

In the present study, the effect of pyriproxyfen was studied on OP-resistant *Cx quinquefasciatus *females that survived a 30-minute exposition to cement-treated surfaces. Cement surfaces were chosen because, being the most porous, they were the only ones that left enough females alive to follow their offspring. Females were exposed to treated surfaces about 36 hours before blood feeding, a timing that would favour a reduction in fecundity over fertility and adult emergence. This is in fact the observation made. For the first nine months, a reduction in fecundity was observed at both doses. A reduction in adult emergence was observed also for nine months but only at the higher dose. An effect on fertility was only observed after treatment and not after nine months. In a recent Phase I evaluation on adult *Anopheles stephensi *females exposed to pyriproxyfen 2% one day after blood feeding, results were the opposite. A reduction in fertility in treatment groups compared to control, whereas fecundity was also reduced but differences failed to be significant [29]. The potential application of horizontal dissemination in malaria vector control needs to be studied [30]. Could the pyriproxyfen that was picked up by females that have survived a prolonged contact with painted walls be then transferred to the oviposition sites of malaria vectors? A project is in progress on different surfaces and blood feeding timing.

Results on non-porous surfaces are satisfying. There is a need to look for ways to deal with the porosity of surfaces like cement. Possible options may include two layers of paint, as discussed above, applying a coating resin, or even natural oil sealers first. The way surfaces are made also makes a difference: cement surfaces can be made less porous depending on the proportion of substances used. Hardwood is more porous than softwood. What would seem clear is that solutions need to be "user-friendly" and appealing in keeping with one of the paint's operational advantages.

There may be a reason to be optimistic about the potential that the insecticide paint may have as an additional tool in malaria and pest control: 1) High long-term killing rates against OP-resistant mosquitoes, 2) IGR's effect on fecundity, fertility and adult emergence and, 3) operational advantages: users can apply the paint themselves and take responsibility for their home improvement.

## Conclusions

Laboratory assays against OP-resistant *Cx. quinquefasciatus *point at the paint's potential in attaining high mortality rates for up to 12 months despite resistance status. Ways to deal with the porosity of certain materials need to be explored. Pyriproxyfen's effect on the fecundity, fertility and adult emergence of exposed adult females affords an added tool in reducing overall pest and malaria vector population densities when the lethal effect of OPs diminishes over time. The paint is easily applied and improves communities' homes. A semi-field study performed following WHOPES Phase II procedures in Benin, West Africa against local populations of pyrethroid-resistant *An. gambiae *and *Cx. quinquefasciatus *populations has confirmed the product's promising profile. Future goals include performing a large-scale entomological, epidemiological and community acceptability study in West Africa.

## Competing interests

The authors declare that they have no competing interests.

## Authors' contributions

PC and SMC conceived the protocol. PC, SMC, SD and BM designed the study. SD, FC and JMH critically contributed to the implementation of the study. BM conducted evaluations. The manuscript has been drafted by BM and has been revised by SMC and SD. All authors read and approved the final manuscript.

## References

[B1] WHO The world malaria report 2005 World Health Organization 2005 Geneva http://rbm.who.int/wmr2005/

[B2] Breman JG Egan A Keusch GT The intolerable burden of malaria: a new look at numbers Am J Trop Med Hyg 2001 64 1-2 iv vii 11425185 10.4269/ajtmh.2001.64.iv

[B3] Snow RW Craig MH Newton C Steketee RW The public health burden of *Plasmodium falciparum *malaria in Africa: deriving the numbers. Disease Control Priorities Project Working Paper No 11 Fogarty International Center, National Institutes of Health 2003 Bethesda, Maryland

[B4] Lengeler C Sharp B Indoor residual spraying and insecticide-treated nets. In: Reducing malaria's burden: evidence of effectiveness for decision makers Global Health Council 2003 Washington D.C 17 24

[B5] Lengeler C Insecticide-treated bed nets and curtains for preventing malaria Cochrane Database of Syst Rev 2008 http://www.cochrane.org 10.1002/14651858.CD000363.pub2 15106149

[B6] Zaim M Aitio A Nakashima N Safety of pyrethroid-treated mosquito nets Med Vet Entomol 2000 14 1 5 10.1046/j.1365-2915.2000.00211.x 10759305

[B7] Baleta A Insecticide resistance threatens malaria control in Africa Lancet 2009 374 1581 1582 10.1016/S0140-6736(09)61933-4 19911449

[B8] Najera JA Zaim M Malaria Vector Control. Insecticides for Indoor Residual Spraying. WHO/CDS/WHOPES/2001.3 World Health Organization 2001 Geneva

[B9] Beier JC Keating J Githure JI Macdonald MB Impoinvil DE Novak RJ Integrated vector management for malaria control Malar J 2008 7 Suppl 1 S4 10.1186/1475-2875-7-S1-S4 19091038 PMC2604879

[B10] United Nations Environment Programme Stockholm Convention on Persistant Organic Pollutants (POPs) UNEP/Convention/The POPs 2010

[B11] Spanish Ministry of Health and Consumer Affairs Report on the study of the toxicity and irritability of Inesfly 5A Health Institute Carlos III 1996 Madrid

[B12] International center of training and medical investigations (CIDEIM) Toxicity studies on Inesfly 5A IGR 2003 CIDEIM, Cali (Colombia) http://www.cideim.org.co

[B13] National Center of Tropical Diseases (CENETROP), Santa Cruz de la Sierra (Bolivia) 2004 http://www.cenetrop.org.bo

[B14] Chandre F Darriet F Doannio JMC Riviere F Pasteur N Guillet P Distribution of organophosphate and carbamate resistance in *Culex pipiens quinquefasciatus *(Diptera: Culicidae) from West Africa J Med Entomol 1997 34 664 671 9439121 10.1093/jmedent/34.6.664

[B15] N'Guessan R Darriet F Guillet P Carnevale P Lamizana MT Corbel V Koffi AA Chandre F Resistance to carbosulfan in field populations of *Anopheles gambiae *from Côte d'Ivoire based on reduced sensitivity of acetylcholinesterase Med Vet Entomol 2003 17 19 25 10.1046/j.1365-2915.2003.00406.x 12680920

[B16] WHO Test Procedures for insecticide resistance monitoring in malaria vectors, bio-efficacy and persistence of insecticides on treated surfaces. Report of the WHO Informal Consultation. Document WHO/CDS/CPC/MAL/1998.12 World Health Organization 1998 Geneva 1 43

[B17] Georghiou GP Metcalf RL Gidden FE Carbamate-resistance in mosquitoes: selection of *Culex pipiens fatigans *Wied (= *Culex quinquefasciatus*) for resistance to Baygon Bull World Health Organ 1966 35 691 708 5297803 PMC2476235

[B18] Berticat C Boquien G Raymond M Chevillon C Insecticide resistance genes induce a mating competition cost in *Culex pipiens *mosquitoes Genetics Research 2002 79 41 47 10.1017/S001667230100547X 11974602

[B19] Najera JA Zaim M Malaria Vector Control. Insecticides for Indoor Residual Spraying. (WHO/CDS/WHOPES/2001.3) World Health Organization 2001 Geneva

[B20] Mosqueira B Chabi J Chandre F Akogbeto M Hougard JM Carnevale P Mas-Coma S Efficacy of an insecticide paint against malaria vectors and nuisance in West Africa. Part II: Field evaluations Malar J 2010 in press 10.1186/1475-2875-9-341 PMC3004939 21108820

[B21] Amelotti I Catalá SS Gorla DE Experimental evaluation of insecticidal paints against *Triatoma infestans *(Hemiptera: Reduviidae), under natural climatic conditions Parasites & Vectors 2009 2 30 36 10.1186/1756-3305-2-30 PMC2714297 19586532

[B22] Rozendaal JA Vector control methods for use by individuals and communities 1997 Geneva: World Health Organization

[B23] Oliveira Filho AM Use of new tools for controlling triatomines in different entomologic situations in the American continent Rev Soc Bras Méd Trop 1996 30 41 46 10.1590/s0037-86821997000100008 9026830

[B24] Itoh T Kawada H Abe A Eshita Y Rongsriyam Y Igarashi A Utilization of bloodfed females of *Aedes aegypti *as a vehicle for the transfer of the insect growth regulator pyriproxyfen to larval habitats J Am Mosq Control Assoc 1994 10 344 347 7807075

[B25] Morrison AC Zielinski-Gutierrez E Scott TW Rosenberg R Defining challenges and proposing solutions for control of the virus vector *Aedes aegypti* PLoS Med 2008 5 e68 10.1371/journal.pmed.0050068 18351798 PMC2267811

[B26] Devine GJ Zamora Perea E Killeen GF Stancil JD Clark SJ Morrison AC Using adult mosquitoes to transfer insecticides to *Aedes aegypti *larval habitats Proc Natl Acad Sci USA 2009 106 11530 11534 10.1073/pnas.0901369106 19561295 PMC2702255

[B27] Dell Chism B Apperson CS Horizontal transfer of the insect growth regulator pyriproxyfen to larval microcosms by gravid *Aedes albopictus *and *Ochlerotatus triseriatus *mosquitoes in the laboratory Med Vet Entomol 2003 17 211 220 10.1046/j.1365-2915.2003.00433.x 12823839

[B28] Sihunincha M Zamora-Perea E Orellana-Rios W Stancil JD Lopez-Sifuentes V Vidal-Ortiz C Devine GJ Potential use of pyriproxyfen for control of *Aedes aegypti *(Diptera: Culicidae) in Iquitos, Peru J Med Entomol 2005 42 620 630 10.1603/0022-2585(2005)042[0620:PUOPFC]2.0.CO;2 16119551

[B29] Aiku AO Yates A Rowland M Laboratory evaluation of pyriproxifen treated bednets on mosquito fertility and fecundity. A preliminary study West Afr J Med 2006 25 22 26 16722354 10.4314/wajm.v25i1.28240

[B30] Devine GJ Killeen GF The potential of a new larviciding method for the control of malaria vectors Malar J 2010 9 142 10.1186/1475-2875-9-142 20500865 PMC2895607

